# A Parting Word

**Published:** 1856-11

**Authors:** H. A. Johnson


### EDITORIAL.

*A Parting Word.*

With the present number, the connection of the undersigned with the *North-Western Medical and Surgical Journal* ceases. With many of our readers we have formed a personal acquaintance which has been to us a source of real pleasure; the names of others, whom we have never seen, have become as familiar as household words. In retiring from the editorial chair and bidding our patrons adieu, we have no apologies to make—we have done what we could, and are content to leave it to our readers to say how well and how faithfully. We confess that our enthusiasm, as a medical journalist, has somewhat abated during the last five years. The sacrifice of time and money is too great: the remuneration too small. A majority of the profession are too poor to pay for what they read, and as the years pass by and their indebtedness increases they become still poorer. We would not insinuate that physicians are dishonest in this matter; but the easiest way to pay the printer is to return a number marked "*refused.*"

We said that our enthusiasm had somewhat abated, and yet we can assure our readers that the *Journal* never had a larger or better paying subscription than it has now, and its prospects for permanence and usefulness were never better than they are to-day. In the hands of Professor Davis, as sole editor and proprietor, we are confident that it will continue to be an efficient instrument for the advancement of the science of medicine, and for elevating the standard of professional education; and we bespeak for him not only the *paying* patronage of all of the present subscribers to the *Journal*, but the assistance and substantial support of the great body of physicians of the North-West.

H. A. JOHNSON.

